# Zooming Into the Microbiota of Home-Made and Industrial Kefir Produced in Greece Using Classical Microbiological and Amplicon-Based Metagenomics Analyses

**DOI:** 10.3389/fmicb.2021.621069

**Published:** 2021-01-28

**Authors:** Maria Kazou, Andriana Grafakou, Effie Tsakalidou, Marina Georgalaki

**Affiliations:** Laboratory of Dairy Research, Department of Food Science and Human Nutrition, Agricultural University of Athens, Athens, Greece

**Keywords:** kefir, grains, microbiological analysis, microbiota, 16S metagenomics analysis, ITS metagenomics analysis, high-throughput sequencing

## Abstract

Kefir is a high nutritional fermented dairy beverage associated with a wide range of health benefits. It constitutes a unique symbiotic association, comprising mainly lactic acid bacteria, yeasts, and occasionally acetic acid bacteria, which is strongly influenced by the geographical origin of the grains, the type of milk used, and the manufacture technology applied. Until recently, kefir microbiota has been almost exclusively studied by culture-dependent techniques. However, high-throughput sequencing, alongside omics approaches, has revolutionized the study of food microbial communities. In the present study, the bacterial, and yeast/fungal microbiota of four home-made samples (both grains and drinks), deriving from well spread geographical regions of Greece, and four industrial beverages, was elucidated by culture-dependent and -independent analyses. In all samples, classical microbiological analysis revealed varying populations of LAB and yeasts, ranging from 5.32 to 9.60 log CFU mL^–1^ or g^–1^, and 2.49 to 7.80 log CFU mL^–1^ or g^–1^, respectively, while in two industrial samples no yeasts were detected. *Listeria monocytogenes*, *Salmonella* spp. and *Staphylococcus* spp. were absent from all the samples analyzed, whereas *Enterobacteriaceae* were detected in one of them. From a total of 123 isolates, including 91 bacteria and 32 yeasts, *Lentilactobacillus kefiri*, *Leuconostoc mesenteroides*, and *Lactococcus lactis* as well as *Kluvyeromyces marxianus* and *Saccharomyces cerevisiae* were the mostly identified bacterial and yeast species, respectively, in the home-made samples. On the contrary, *Streptococcus thermophilus, Lactobacillus delbrueckii* subsp. *bulgaricus*, and *Lacticaseibacillus rhamnosus* along with *Debaryomyces hansenii* and *K. marxianus* were the main bacterial and yeast species, respectively, isolated from the industrial beverages. In agreement with the identification results obtained from the culture-dependent approaches, amplicon-based metagenomics analysis revealed that the most abundant bacterial genera in almost all home-made samples (both grains and drinks) were *Lactobacillus* and *Lactococcus*, while *Saccharomyces, Kazachstania*, and *Kluvyeromyces* were the predominant yeasts/fungi. On the other hand, *Streptococcus, Lactobacillus*, and *Lactococcus* as well as *Kluvyeromyces* and *Debaryomyces* dominated the bacterial and yeast/fungal microbiota, respectively, in the industrial beverages. This is the first report on the microbiota of kefir produced in Greece by a holistic approach combining classical microbiological, molecular, and amplicon-based metagenomics analyses.

## Introduction

At both research and industrial level, dairy fermented foods are the protagonists among functional foods, i.e., foods having a positive impact on human health beyond the known nutritional value, such as benefits concerning gastrointestinal health, hypertension, cholesterol reduction, immune system regulation, interaction between gut and brain, etc. (Shiby and Mishra, 2013). Fermented milks, with yogurt standing as the main representative, are probably the most prominent among them, offering a wide range of appealing sensory characteristics and well-being benefits (Robinson and Itsaranuwat, 2006). Other fermented milks, such as kefir, koumiss, chigo, viili, nunu, amabere, amaruranu, and suusac (Ishii et al., 1997; Lore et al., 2005; Kahala et al., 2008; Akabanda et al., 2013; Nyambane et al., 2014; Yao et al., 2017) are known as alcoholic milk beverages, since milk is fermented by both lactic acid bacteria (LAB) and yeasts (yeast-lactic fermentation) (Wszolek et al., 2006).

Kefir, a name most likely deriving from the Turkish and Caucasian word keyif meaning pleasure (Kurman et al., 1992; Kabak and Dobson, 2011), is one of the most cherished functional dairy products. It is believed to have its origins in the Caucasian, Tibetan and Mongolian mountains, but is also manufactured artisanally for centuries in several countries, such as the former Union of Soviet Socialist Republics (USSR), Bulgaria, Slovakia, Hungary, Portugal, Turkey, and France (Farnworth, 2005; Wszolek et al., 2006). In recent years, kefir consumption has increased worldwide, and by 2023, the global kefir market is expected to reach 1.85 billion $US (Shahbandeh, 2019). This can be attributed not only to its unique sensory characteristics, such as flavor, aroma, freshness, and viscosity, but also to a plethora of bioactive compounds, such as peptides and vitamins (Farnworth, 2005; Kabak and Dobson, 2011; Leite et al., 2012, 2013; Diosma et al., 2014). Consumption of kefir has been associated with numerous health benefits including anticarcinogenic and anti-inflammatory effects (Rodrigues et al., 2005; de Moreno de Leblanc et al., 2007; Lee et al., 2007; Kim et al., 2016), alleviation of lactose intolerance symptoms and cholesterol reduction (Hertzler and Clancy, 2003; Liu et al., 2006).

This viscous, slightly carbonated dairy beverage comprises a complex microbial association of mainly LAB, acetic acid bacteria (AAB) and yeasts. When LAB and yeasts are used as starters for the industrial production of kefir beverage, the respective counts in the final product should be at least 7 and 4 log CFU mL^–1^, until the “date of minimum durability” (Codex Alimentarius Commission, 2003). In kefir symbiotic ecosystem, LAB ferment lactose and decrease the pH, while yeasts stimulate LAB growth by producing B-group vitamins and hydrolyzing milk proteins (Farnworth, 2005; Álvarez-Martín et al., 2008). This symbiosis results in an elastic, white to yellow and slimy granules with irregular, cauliflower-like structure of different size with folded or uneven surface, called kefir grains, which also comprise coagulated milk proteins and mucous polysaccharides known as kefiran (Farnworth, 2005; Wszolek et al., 2006; Dobson et al., 2011; Kabak and Dobson, 2011; Leite et al., 2013). However, kefir beverage can be also produced artisanally by the “Russian method,” i.e., by fermenting milk with kefir grains. At the end of the fermentation the grains are removed and stored for subsequent use (Kabak and Dobson, 2011; Prado et al., 2015). Nevertheless, at the end of the fermentation, kefir beverage has a typical pH value of 4.5–4.6 (Guzel-Seydim et al., 2005).

The microbial composition of kefir grains and beverages has been well documented by culture-dependent and -independent fingerprinting methods, mainly PCR-denaturing gradient gel electrophoresis (PCR-DGGE) (Pintado et al., 1996; Witthuhn et al., 2005; Chen et al., 2008; Miguel et al., 2010; Kesmen and Kacmaz, 2011; Leite et al., 2013; Diosma et al., 2014). However, the development and application of high-throughput sequencing (HTS), alongside omics approaches, fundamentally altered the way of studying food microbial consortia, highlighting also the impact of various factors, such as the type of milk, the geographical origin and the manufacture method on kefir microbiota (Dobson et al., 2011; Leite et al., 2012; Gao et al., 2013; Marsh et al., 2013; Nalbantoglu et al., 2014; Garofalo et al., 2015; Korsak et al., 2015; Bourrie et al., 2016; Zamberi et al., 2016; Dertli and Con, 2017). Nowadays, amplicon-based metagenomics analysis targeting either the 16S rRNA gene or the internal transcribed spacers (ITS) DNA region is the most widely used technique to analyze the bacterial and yeasts/fungal communities, respectively, in a food matrix (De Filippis et al., 2017; Ferrocino and Cocolin, 2017).

The aim of the present study was to elucidate and compare the bacterial and yeast/fungal microbiota of home-made and industrial kefir samples produced in Greece, deriving from well-spread geographical origin and types of milk, using a dual approach that includes both classical microbiological and amplicon-based metagenomics analyses.

## Materials and Methods

### Samples

Four home-made kefir grains (G) and the respective drinks (D) deriving from well spread geographical regions of Greece, as well as four industrially produced kefir beverages (no grains present) were analyzed in this study (Table 1). Home-made samples were prepared from commercial pasteurized milk fermented with home-maintained grains (kept in milk or milk and tap water for 7–20 days at 4°C) for 18–24 h, at ambient temperature (25–30°C), while industrial samples were prepared from pasteurized milk fermented with commercial kefir starters. pH was measured using a digital pH meter (model 827 pH lab; Metrohm, Herisau, Switzerland).

**TABLE 1:** Kefir samples examined.

Sample*	**Production**	**Geographical origin**	**Milk type**	**pH**
1G	Home-made	Athens, Central Greece	Cow and Goat (1:1)	3.43
1D	Home-made	Athens, Central Greece	Cow and Goat (1:1)	4.17
2	Industrial	Kilkis, Northern Greece	Cow	4.40
3	Industrial	Pella, Northern Greece	Goat	3.72
4	Industrial	Sindos, Northern Greece	Buffalo	4.49
5	Industrial	Volos, Central Greece	Goat	4.24
6G	Home-made	Ioannina, North-western Greece	Cow	4.54
6D	Home-made	Ioannina, North-western Greece	Cow	4.37
7G	Home-made	Gythio, Southern Greece	Cow	5.18
7D	Home-made	Gythio, Southern Greece	Cow	5.95
8G	Home-made	Karditsa, Central Greece	Cow	3.90
8D	Home-made	Karditsa, Central Greece	Cow	3.80

*G and D denote grain and drink kefir samples, respectively.

### Microbiological Analysis

The following groups of microorganisms were enumerated in both kefir grains and drinks: (1) thermophilic LAB in MRS agar (pH 6.2; Biokar Diagnostics, Beauvais, France) at 37°C for 3 days; (2) mesophilic LAB in MRS agar at 30°C for 3 days; (3) non-starter LAB (NSLAB) on Rogosa agar (Biokar) at 30°C for 5 days, anaerobically (double agar layer); (4) enterococci on kanamycin aesculin azide (KAA) agar (Merck, Darmstadt, Germany) at 37°C for 1 day; (5) AAB on medium called GYP, containing 1% w/v glucose (PanReac AppliChem, Darmstadt, Germany), 0.8% w/v yeast extract (Biokar), 1.5% w/v bacteriological peptone (Sigma-Aldrich, St. Louis, US) and 1.5% w/v agar (Condalab, Madrid, Spain) at 30°C for 2 days; (6) yeasts and molds on yeast extract glucose chloramphenicol (YGC) agar (Merck) at 25°C for 3–4 days; (7) Enterobacteriaceae on violet red bile glucose (VRBG) agar (Biokar) at 37°C for 1 day, anaerobically (double agar layer); (8) *Listeria monocytogenes* on Harlequin Listeria Chromogenic Agar (LabM) at 30°C for 1 day; (9) *Salmonella* spp. on xylose lysine deoxycholate (XLD) agar (LabM, Heywood, United Kingdom) at 37°C for 1 day; and (10) micrococci-staphylococci on mannitol salt agar (MSA) (Biokar) at 37°C for 1 day. Cycloheximide (Merck) was added (150 μg mL^–1^) to media 1, 2, 3, and 5, in order to eliminate growth of yeasts (Camu et al., 2008). Results were expressed as log CFU g^–1^ (grains) or mL^–1^ (beverages).

Based on morphology (shape, color, and size), colonies were collected from MRS (37 and 30°C), Rogosa, GYP, and YGC agar plates, purified by repetitive streaking, and identified at the species level.

### DNA Extraction and Rep-PCR Fingerprinting

Genomic DNA of bacterial isolates was extracted from a 2 mL overnight culture as described previously (Georgalaki et al., 2017). In addition, DNA extraction from yeast cells grown overnight in medium containing 5% w/v yeast extract and 20% w/v glucose was performed according to the protocol of Kopsahelis et al. (2009), with some modifications in two steps. First, cell pellet was washed twice with dd H_2_O and incubated at 65°C for 10 min before lysis to decrease the content of PCR inhibitors, and second (last protocol steps), DNA pellet was washed with iced-cold 70% v/v ethanol and re-suspended in a small volume (30–50 μL) of Tris-EDTA (TE) buffer (10 mM Tris-HCl, 1 mM EDTA, pH 8). DNA concentration of both bacteria and yeast isolates was measured using a Quawell Q5000 Read First photometer (Quawell Technology Inc., San Jose, CA, United States).

Repetitive extragenic palindromic elements-PCR (rep-PCR) analysis of bacteria isolates was performed according to Georgalaki et al. (2017). For yeasts, a slightly modified protocol of da Silva-Filho et al. (2005) was employed. In details, amplification was performed in 25 μl PCR reaction volume containing 50 ng DNA, 0.3 mM (GTG)_5_ primer (5′-GTG GTG GTG GTG GTG-3′; VBC Biotech, Vienna, Austria) and 12.5 μl OneTaq-Quick Load 2× Master Mix (New England Biolabs, MA, United States). PCR was performed using a SimpliAmp Thermal Cycler (Thermo Fisher Scientific, Waltham, MA, United States) as follows: initial denaturation at 94°C for 5 min, 35 cycles with denaturation at 94°C for 15 s, primer annealing at 55°C for 45 s and primer extension at 72°C for 90 s, followed by a final extension at 72°C for 15 min.

Bacteria and yeast rep-PCR products were electrophoretically separated and the BioNumerics version 6.0 (Applied Maths, Ghent, Belgium) was used for rep-PCR fingerprints clustering (Georgalaki et al., 2017).

### Bacteria and Yeasts Identification

Representative bacterial and yeast isolates based on the clustering of the rep-PCR analysis were identified at the species level by sequencing the 16S rRNA gene and the ITS DNA region using the primers 16SF (5′-GGA GAG TTA GAT CTT GGC TCA G-3′)/16SR (5′-AGA AAG GAG GTG ATC CAG CC-3′) (Ntougias et al., 2006) and ITS1 (5′-TCC GTA GGT GAA CCT TGC GG-3′)/ITS4 (5′-TCC TCC GCT TAT TGA TAT GC-3′) (Korabecna, 2007), respectively. After electrophoresis, PCR products were purified using the NucleoSpin^®^ Gel and PCR Clean-up kit (Macherey-Nagel, Duren, Germany).

### Amplicon-Based Metagenomics Analysis

#### Total DNA Extraction and Amplicon Sequencing

Microbial DNA from the kefir grains and beverages (home-made and industrial ones) was extracted using the DNeasyPowerSoil Kit (Qiagen, Valencia, CA, United States) and the DNeasyPowerFood Microbial Kit (Qiagen), respectively, according to the manufacturer’s instructions. DNA was eluted in 30 μL of preheated (70°C) DNA-free PCR grade water and stored at −20°C until amplicon sequencing. DNA concentration and quality were determined using a Quawell Q5000 Read First photometer (Quawell Technology Inc.).

Amplicon sequencing (bTEFAP) was performed on the Illumina MiSeq sequencing platform at Molecular Research DNA (MR DNA, Shallowater, Texas). Bacterial diversity was evaluated through the amplification of the V1–V3 hypervariable region of the 16S rRNA gene using primers 27F (5′-AGR GTT TGA TCM TGG CTC AG-3′) and 519R (5′-GTN TTA CNG CGG CKG CTG-3′). On the other hand, primers ITS1F (5′-CTT GGT CAT TTA GAG GAA GTA A-3′) and ITS2R (5′-GCT GCG TTC TTC ATC GAT GC-3′) were used to amplify part of the ITS DNA region of yeasts/fungi, namely ITS1-ITS2. The PCR conditions and purification of amplicon products were performed according to Papademas et al. (2019). Operational taxonomic units (OTUs) were defined after removal of singleton sequences, clustered at 97% similarity and taxonomically assigned using the Nucleotide Basic Local Alignment Search Tool (BLASTn) against a curated National Center for Biotechnology Information (NCBI) deriving database (Dowd et al., 2008). Raw sequencing data are deposited at the European Nucleotide Archive (ENA) under the study PRJEB37688.

#### Alpha-and Beta-Diversity Analysis

Bacterial and yeast/fungal microbiota was analyzed in R version 3.6.3 using the R packages phyloseq and ggplot2, as well as several custom packages (Poirier et al., 2018). Therefore, one bacterial and one yeast/fungal phyloseq object was created, each containing three files, namely otu_table, in which the OTU abundances were normalized using the median sequencing depth of all samples analyzed, taxa_table and sample_data. These two datasets were used for all downstream analyses.

Alpha-diversity analysis was calculated according to the Observed species and the Shannon and inverse Simpson indexes for species diversity according to the abundance and uniformity of OTUs (Thukral, 2017). Analysis of variance (ANOVA) was performed to determine whether the differences among the samples were statistically significant, using a threshold value of 0.05. Moreover, clustered OTUs were used to construct the rarefaction curves in order to assess species richness and estimate the sequencing depth.

In addition, beta-diversity analysis was conducted to examine similarities and dissimilarities among the different food samples based on the bacterial and yeast/fungal microbiota. Thus, a MultiDimensional Scaling (MDS) Principle Coordinates Analysis (PCoA) based on Bray-Curtis distance metrics and a hierarchical clustering of Bray-Curtis using ward.d2 were performed on the bacterial and yeast/fungal communities taxonomically assigned at the family level (Whittaker, 1972).

## Results and Discussion

### Classical Microbiological Analysis

Results obtained through the classical microbiological analysis are summarized in Table 2. In general, high populations of presumptive mesophilic and thermophilic LAB were counted in all kefir samples, no matter home-made, industrial, geographical origin, type of milk or pH of the product. More specifically, counts ranged from 6.50 to 9.60 log CFU mL^–1^ or g^–1^ (mesophilic LAB, home-made samples), from 6.60 to 9.20 log CFU mL^–1^ or g^–1^ (thermophilic LAB, home-made samples), from 6.38 to 9.15 log CFU mL^–1^ (mesophilic LAB, industrial samples) and from 5.32 to 8.60 log CFU mL^–1^ (thermophilic LAB, industrial, samples). Concerning the home-made samples, no differences were observed between grains and drinks in samples 6 and 8, while in samples 1 and 7, counts in grains were by 1–2 log higher than those in the respective drinks. This can be probably attributed to microbial aggregation and/or biofilm formation in the kefir grains resulting in a variability of biodiversity between grains and beverages, as well as to the variable microbial communities within the grain layers (Dobson et al., 2011; Wang et al., 2012). Accordingly, similar or even higher LAB populations than those of the present study have been reported in previous studies for kefir grain and their respective drink samples (Pintado et al., 1996; Guzel-Seydim et al., 2005; Kesmen and Kacmaz, 2011; Garofalo et al., 2015; Korsak et al., 2015). However, no significant differences were observed between grains and drinks (Guzel-Seydim et al., 2005; Kesmen and Kacmaz, 2011). Interestingly, the lowest LAB values were obtained with the industrial samples 4 and 5, namely 6.38 and 6.62 log CFU mL^–1^ at 30°C, and 5.32 and 6.02 log CFU mL^–1^ at 37°C, respectively. These results showed that only the industrial samples 2 and 3 complied with the 2003 Codex Alimentarius standard (7 log CFU mL^–1^).

**TABLE 2:** Microbial counts (log CFU g^–1^ or mL^–1^ ± *SD*) in the kefir samples examined.

	Home-made samples*			
	
	**1G**	**1D**	**6G**	**6D**
Thermophilic LAB (MRS agar, 37°C)	8.43 ± 0.02	7.29 ± 0.08	8.67 ± 0.02	8.60 ± 0.02
Mesophilic LAB (MRS agar, 30°C)	8.47 ± 0.13	7.27 ± 0.06	8.75 ± 0.04	8.54 ± 0.08
NSLAB (Rogosa agar, 30°C)	8.42 ± 0.04	6.11 ± 0.02	8.74 ± 0.17	8.56 ± 0.04
Enterococci (KAA agar, 37°C)	nd	nd	6.60 ± 0.03	5.60 ± 0.21
AAB (GYP agar, 30°C)	7.40 ± 0.08	6.11 ± 0.19	8.67 ± 0.02	7.78 ± 0.04
Yeasts and molds (YGC agar, 30°C)	6.61 ± 0.18	6.11 ± 0.08	6.32 ± 0.09	5.67 ± 0.01
*Enterobacteriaceae* (VRBG agar, 37°C)	nd	nd	1.00 ± 0.08	1.48 ± 0.03
*Listeria monocytogenes* (HALO10 agar, 30°C)	nd	nd	nd	nd
*Salmonella* spp. (XLD agar, 30°C)	nd	nd	nd	nd
*Staphylococcus* spp. (MSA agar, 37°C)	nd	nd	nd	nd

	Home-made samples*			
	
	**7G**	**7D**	**8G**	**8D**

Thermophilic LAB (MRS agar, 37°C)	9.20 ± 0.04	7.80 ± 0.08	6.60 ± 0.09	6.60 ± 0.29
Mesophilic LAB (MRS agar, 30°C)	9.60 ± 0.13	7.60 ± 0.02	6.50 ± 0.09	6.50 ± 0.02
NSLAB (Rogosa agar, 30°C)	8.40 ± 0.05	7.00 ± 0.09	7.60 ± 0.08	7.60 ± 0.06
Enterococci (KAA agar, 37°C)	nd	3.48 ± 0.05	0.00	0.00
AAB (GYP agar, 30°C)	6.40 ± 0.09	8.50 ± 0.02	7.10 ± 0.02	7.10 ± 0.03
Yeasts and molds (YGC agar, 30°C)	7.80 ± 0.05	5.70 ± 0.12	6.10 ± 0.05	6.10 ± 0.03
*Enterobacteriaceae* (VRBG agar, 37°C)	3.50 ± 0.02	6.50 ± 0.07	nd	nd
*Listeria monocytogenes* (HALO10 agar, 30°C)	nd	nd	nd	nd
*Salmonella* spp. (XLD agar, 30°C)	nd	nd	nd	nd
*Staphylococcus* spp. (MSA agar, 37°C)	nd	nd	nd	nd

	**Industrial samples**			
	
	**2**	**3**	**4**	**5**

Thermophilic LAB (MRS agar, 37°C)	8.03 ± 0.02	8.60 ± 0.02	5.32 ± 0.02	6.02 ± 0.05
Mesophilic LAB (MRS agar, 30°C)	9.15 ± 0.08	8.60 ± 0.07	6.38 ± 0.03	6.62 ± 0.10
NSLAB (Rogosa agar, 30°C)	8.01 ± 0.12	8.33 ± 0.13	6.41 ± 0.09	6.76 ± 0.02
Enterococci (KAA agar, 37°C)	nd	nd	2.48 ± 0.02	0.81 ± 0.07
AAB (GYP agar, 30°C)	8.01 ± 0.02	8.60 ± 0.02	6.77 ± 0.05	8.24 ± 0.02
Yeasts and molds (YGC agar, 30°C)	nd	nd	2.49 ± 0.08	5.63 ± 0.09
*Enterobacteriaceae* (VRBG agar, 37°C)	nd	nd	0.60 ± 0.02	1.40 ± 0.12
*Listeria monocytogenes* (HALO10 agar, 30°C)	nd	nd	nd	nd
*Salmonella* spp. (XLD agar, 30°C)	nd	nd	nd	nd
*Staphylococcus* spp. (MSA agar, 37°C)	nd	nd	nd	nd

*G and D denote grain and drink kefir samples, respectively; nd, not detected; Values are the mean ± SD (n = 3).

Furthermore, high populations of NSLAB were also counted in all kefir samples, ranging from 6.11 to 8.56 log CFU mL^–1^ (home-made kefir drinks), from 7.60 to 8.74 log CFU g^–1^ (home-made kefir grains) and from 6.41 to 8.33 log CFU mL^–1^ (industrial samples). As in the case of mesophilic and thermophilic LAB, no differences between grains and drinks were observed in samples 6 and 8, while, again, in samples 1 and 7 counts in grains were higher than those in the respective drinks. Similarly, the lowest NSLAB counts were obtained in the industrial samples 4 and 5. There are no reports on the population of NSLAB in kefir using Rogosa agar, although enumeration of kefir mesophilic lactobacilli and cocci using MRS and M17 agar, respectively, resulted in similar counts (Guzel-Seydim et al., 2005; Kesmen and Kacmaz, 2011).

Enterococci were detected in two out of the four home-made samples, namely 6 and 7, in both grains and drinks for sample 6 (6.60 log CFU g^–1^ in 6G, and 5.60 log CFU mL^–1^ in 6D), but only in the drink of sample 7 (7D; 3.48 log CFU mL^–1^). Moreover, they were detected in two out of the four industrial samples, namely 4 and 5 at rather low counts of 2.48 and 0.81 log CFU mL^–1^, respectively. To the best of our knowledge, no enterococci enumeration in kefir using KAA agar has been reported so far. However, *Enterococcus faecalis* and *Enterococcus durans* have been isolated from Tibetan kefir beverages (Yang et al., 2007), *E. durans* has been isolated from kefir grains belonging to the Centro de Investigación y Desarrollo en Criotecnología de Alimentos (CIDCA) collection (Carasi et al., 2014), while *Enterococcus* spp. isolates have been identified in home-made or commercial kefir grains on M17 agar (Garofalo et al., 2015). Thus, the presence of enterococci in kefir is mostly ambiguous. It should be stressed that although enterococci comprise a significant part of many fermented foods, they are, at the same time, considered as indicators of poor hygienic processing conditions, with strains of some species exhibiting virulence factors (Foulquié Moreno et al., 2006).

Moreover, high microbial counts on GYP agar plates (dedicated for AAB) were observed, with values ranging between 6.40 and 8.67 log CFU g^–1^ for grain samples, and 6.11 and 8.60 log CFU mL^–1^ for drink samples, either home-made or industrial. However, isolates from the GYP agar plates were found to be either Gram-positive bacteria or yeasts. Problematic cultivation and enumeration of AAB from natural environments has been already reported (Camu et al., 2008; Gulitz et al., 2011). Some authors assign this to the viable but non-culturable state switch of AAB under not favorable growth media and conditions (Gomes et al., 2018). Thus, while Pintado et al. (1996) and Witthuhn et al. (2005) reported the absence of AAB in Portuguese and African kefir grains, respectively, Garofalo et al. (2015), using three different growth media (all containing cycloheximide at 400 μg mL^–1^), reported AAB viable counts in different grain samples and of different origin, ranging from 3 to 6 log CFU g^–1^.

Yeasts were detected in all home-made samples, both grains and drinks, with counts ranging from 6.10 to 7.80 log CFU g^–1^ (grain samples) and from 5.67 to 6.11 log CFU mL^–1^ (drink samples). On the other hand, they were only detected in two out of the four commercial samples, namely 4 and 5, at 2.49 and 5.63 log CFU mL^–1^, respectively. Consequently, only the commercial sample 5 met the Codex Alimentarius standard for yeasts. Similar counts, ranging between 5.0 and 6.55 log CFU g^–1^ or mL^–1^, were reported by Korsak et al. (2015) for grains, either home-made or found in the market, and by Guzel-Seydim et al. (2005) for grains and drinks produced at a Turkish University, whereas Garofalo et al. (2015) reported yeast populations at 7 log CFU g^–1^ for grain samples produced by various suppliers. Additionally, Kalamaki and Angelidis (2016) reported that in kefir samples produced in Greece, yeasts were counted at 7.7 log CFU g^–1^ in grains and from < 0.4 to 6.7 log CFU mL^–1^ in drinks. In all cases however, yeast populations were lower than the mesophilic and thermophilic LAB.

*Enterobacteriaceae* were detected in two of the four home-made samples, namely 6 and 7, in both grains (1.0 and 3.5 log CFU g^–1^, respectively), and drinks (1.48 and 6.5 log CFU mL^–1^, respectively). Interestingly, *Enterobacteriaceae* population of the home-made sample 7D was higher (3 log difference) than the respective grain sample 7G, which indicates the advantageous milk environment for the growth of this bacterial group. They were also detected in two of the four industrial samples, namely 4 and 5, at low levels of 0.6 and 1.4 log CFU mL^–1^, respectively, though. Samples with > 0.7 log CFU mL^–1^ do not fulfill the above-mentioned Commission Regulation microbiological criteria for liquid dairy products, which requires presence of *Enterobacteriaceae* < 0.7 log CFU mL^–1^. The detection of both enterococci and *Enterobacteriaceae* in the same industrial samples, namely 4 and 5, as well as home-made samples 6 and 7, could be probably attributed to deficient hygienic handling and storage conditions. Detection of *Enterobacteriaceae* has been reported by Chen et al. (2008) who isolated an *Escherichia coli* strain from Taiwanese kefir grains and attributed its occurrence to possible environmental contamination.

Finally, no growth was observed on the media used for *L. monocytogenes, Salmonella* spp., and *Staphylococcus* spp., and in any of the samples examined; thus all samples fulfilled the microbiological criteria of the Commission Regulation (2005) No 2073/2005.

### Strain Fingerprinting and Identification of Microbial Isolates

Based on colony morphology, a total of 123 isolates, including 91 bacteria and 32 yeasts, were selected from the MRS (37 and 30°C), Rogosa, GYP, and YGC agar plates. Rep-PCR analysis clustered bacteria in 27 and yeasts in 10 groups. Representative isolates of all groups were selected and subjected to 16S rRNA gene and ITS DNA region sequencing, respectively.

According to the sequencing results (Supplementary Table S1A and Supplementary Figure S1), among the 91 bacterial isolates, 31 isolates were identified as *Lentilactobacillus kefiri* (basonym *Lactobacillus kefiri*), 16 as *Leuconostoc mesenteroides*, 12 as *Lacticaseibacillus rhamnosus* (basonym *Lactobacillus rhamnosus*), eight as *Streptococcus thermophilus*, seven as *Lactococcus lactis*, seven as *Leuconostoc mesenteroides/pseudomesenteroides*, four as *Enterobacter clocae/ludwigii/kobei*, two as *Staphylococcus warneri*, one as *L. delbrueckii* subsp. *bulgaricus*, one as *Lactococcus raffinolactis*, one as *Streptococcus parauberis*, and one as *Klebsiella oxytoca.* Interestingly, all bacterial species used as starters for the production of industrial sample 4 were isolated and identified (*L. lactis*, *S*.thermophilus, and *Leuconostoc* sp.), while information on the starters used for the rest of the samples was not available. Additionally, *L. kefiri* was found exclusively in home-made samples where it is considered the dominant species (62.0%) along with *L. mesenteroides/pseudomesenteroides* (30.0%), whereas *S*. thermophilus was only found in the industrial ones. The dominant species identified in the industrial samples were *L. rhamnosus* (29.3%), *S. thermophilus* (22.0%), and *L. mesenteroides/pseudomesenteroides* (19.5%) depending on the sample analyzed. Interestingly, the eight isolates belonged to species, which are considered (opportunistic) pathogens, i.e., *S. warneri, E. clocae/ludwigii/kobei, S. parauberis*, and *K. oxytoca* were all isolated from the home-made sample 7 (the *S. warneri* from the grains and the other from the drink), which indicates poor hygiene practices in the production of sample 7. The presence of the bacterial species identified has been routinely reported in kefir (Pintado et al., 1996; Chen et al., 2008; Miguel et al., 2010; Kesmen and Kacmaz, 2011). However, to the best of our knowledge, this is the first time that the above mentioned (opportunistic) pathogens are detected in kefir samples.

Among the 32 yeast isolates (Supplementary Table S1B and Supplementary Figure S2), 14 were identified as *Kluvyeromyces marxianus*, nine as *Saccharomyces cerevisiae*, three as *Kazachstania turicensis*, two as *Geotrichum candidum/Galactomyces candidum*, two as *Yarrowia lipolytica*, one as *Pichia kudriavzevii*, and one as *Debaryomyces hansenii. K. marxianus* and *S. cerevisiae* were the dominant species in the home-made samples, while four out of the six isolates of the industrial samples belonged to *K. marxianus* and *Geotrichum candidum/Galactomyces candidum*. Interestingly, no yeasts were obtained from the industrial samples 2 and 3, while *D. hansenii*, which was the yeast species used as starter for the production of industrial sample 4 was isolated and identified. *K. marxianus* and *S. cerevisiae* are considered as the predominant species in kefir (43.8 and 28.1%, respectively). *K. marxianus* and *K. turicensis* play an important role in kefir grain formation (Wang et al., 2012), *Geotrichum candidum* can be found at the early stages of kefir production and it covers the grain surface (Witthuhn et al., 2005), while *Y. lipolytica* and some *Pichia* species are of significant importance in the production of fermented milks, such as kefir and koumiss (Fleet, 2006). Similar results have been reported in kefir samples from Argentine (Diosma et al., 2014), Italy (Garofalo et al., 2015) and Africa (Witthuhn et al., 2005).

### Amplicon-Based Metagenomics Analysis

#### Sequencing Data of Microbial Communities in Kefir Grains and Beverages

A total of 1,061,686 bacterial raw sequences were obtained from the 12 samples analyzed, i.e., four kefir grains (samples 1G, 6G, 7G, and 8G) and the respective home-made drinks (samples 1D, 6D, 7D, and 8D) as well as four industrial beverages (samples 2, 3, 4, and 5). After the quality control of the 16S reads, 666,437 sequences were used for taxonomic classification, with an average of 55,536 ± 9,374 sequences per sample. In total, 160 bacterial OTUs were assigned among the samples. Interestingly, the average number of OTUs was similar among the three food sample groups, i.e., 73 ± 22, 75 ± 12, and 68 ± 17 OTUs per kefir grain, home-made and industrial drinks, respectively. On the contrary, the number of yeast/fungal raw sequences obtained from the 12 samples was higher compared to that of bacterial sequences, i.e., 1,413,675, as well as the number of sequences that passed the quality control, i.e., 938,653, with an average of 78,221 ± 52,180 sequences per sample. Among the 12 samples analyzed, 463 yeast/fungal OTUs were identified, ranging from 113 (sample 6D) to 301 (sample 2) OTUs, with an average of 148 ± 12, 149 ± 25, and 250 ± 58 OTUs per kefir grain, home-made, and industrial drinks, respectively.

#### Alpha-and Beta-Diversity Analysis

**FIGURE 1:**
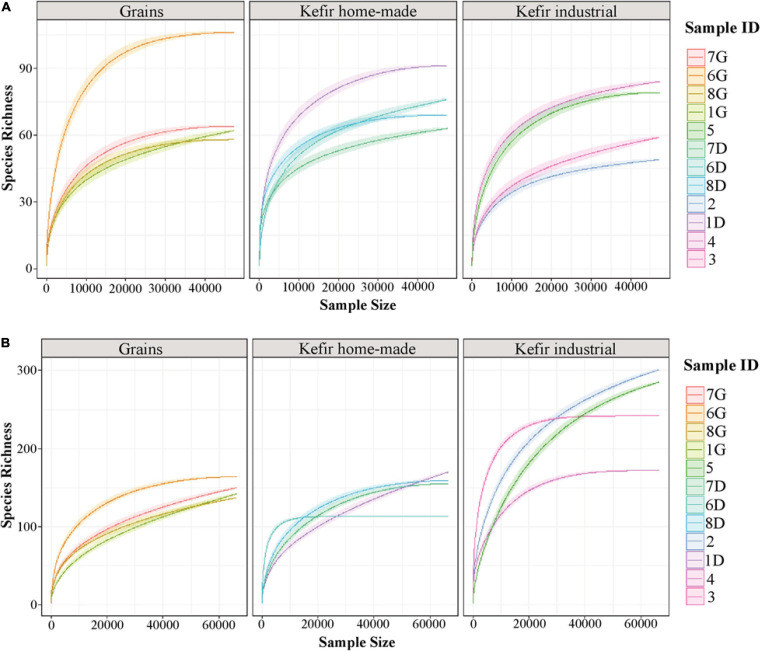
Rarefaction curves of species richness showing the sequencing depth of 16S **(A)** and ITS **(B)** data obtained from kefir grains (samples 1G, 6G, 7G, and 8G), home-made (samples 1D, 6D, 7D, and 8D) and industrial (samples 2, 3, 4, and 5) drinks. The rarefaction curves for each sample were displayed by different colors. The *x*-axis represents the sequencing depth in number of reads and the *y*-axis the estimation of the OTU richness detected at species level.

The rarefaction curve analysis was performed to evaluate the sufficiently recovered OTUs by the Illumina MiSeq sequencing. Rarefaction curves of both 16S and ITS data of the majority of the samples analyzed attained the saturation plateau, indicating that the sequencing depth was sufficient (Figure 1). It should be noted though, that rarefaction curves of the ITS data for kefir grains 1G, 7G, and 8G, home-made drink 1D and industrial drinks 2 and 5, did not tend to approach the saturation plateau, indicating that the yeast/fungal richness in these samples was probably underestimated (Figure 1B).

**FIGURE 2:**
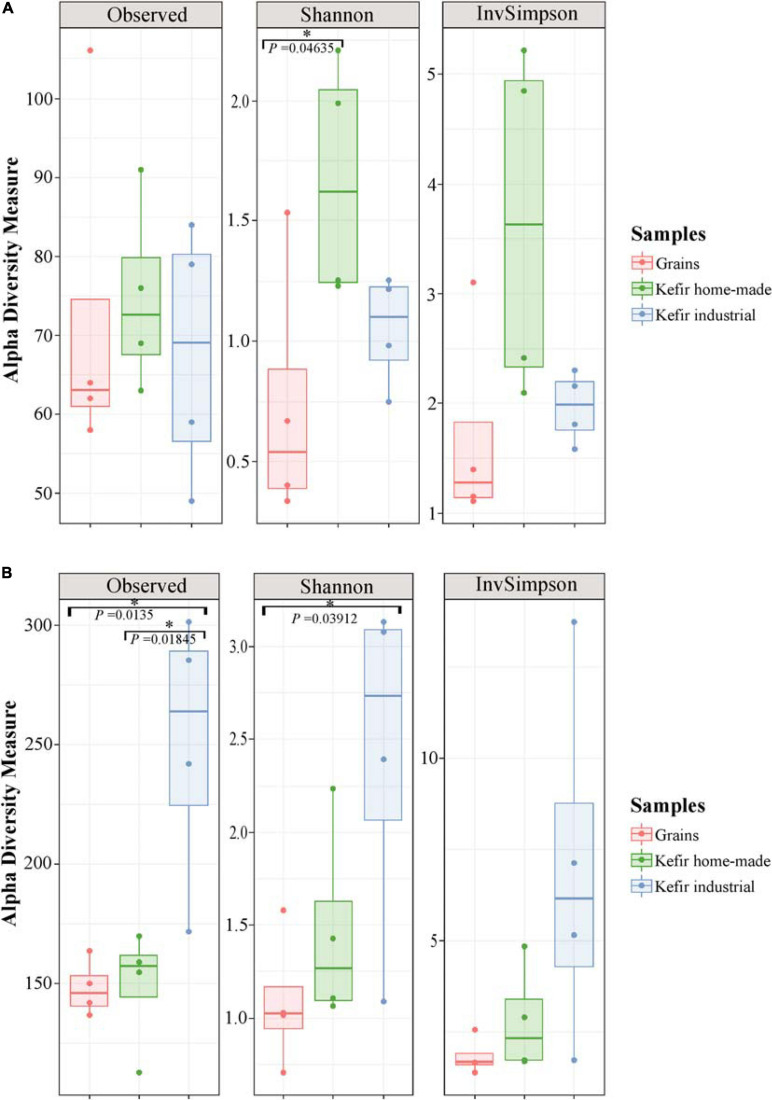
Boxplots of alpha-diversity indices, namely Observed, Shannon, and inverse Simpson for bacterial **(A)**, and yeast/fungal **(B)** communities identified in kefir grains, home-made, and industrial drinks. Samples are colored according to the different food groups, i.e., red for kefir grains, green for home-made drinks and blue for industrial beverages. An asterisk denoted *P* < 0.05.

The microbial complexity (richness and evenness) was estimated on the basis of alpha-diversity indices, namely Observed, Shannon, and inverse Simpson. The richness estimation according to Observed species, indicated that the yeast/fungal microbiota of industrial beverages was significantly higher (*P* < 0.05) compared to that of home-made drinks and kefir grains, while the species richness of bacterial communities did not differ significantly among the samples (Figure 2). The microbial richness based on Observed species was strongly supported also by the rarefaction curves analysis, as mentioned above (Figure 1). On the other hand, a significant difference (*P* < 0.05) was observed in Shannon and inverse Simpson indices of diversity, in both bacterial and yeast/fungal communities among the samples (Figure 2). In details, bacterial microbiota of home-made beverages was found to be the most abundant, followed by that of industrial drinks and kefir grains (Figure 2A). However, this was not the case for yeast/fungal microbiota, since the abundance of industrial beverages was higher than that of home-made drinks and kefir grains (Figure 2B).

**FIGURE 3:**
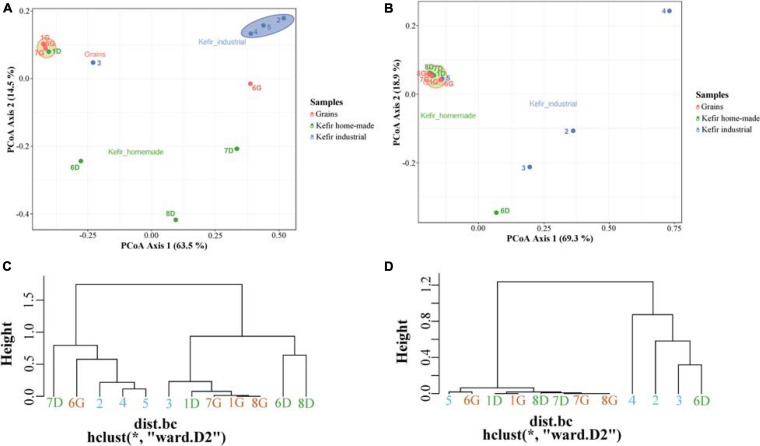
Principal coordinates analysis (PCoA) among bacterial **(A)** and yeasts/fungal **(B)** communities found in kefir grains (samples 1G, 6G, 7G, and 8G), home-made (samples 1D, 6D, 7D, and 8D) and industrial (samples 2, 3, 4, and 5) drinks. Hierarchical clustering of bacterial **(C)** and yeasts/fungal **(D)** communities identified in kefir grains, home-made, and industrial drinks. All OTUs in both PCoA and hierarchical clustering within the communities were taxonomically assigned at the family level. Samples are colored according to the different food groups, i.e., red for kefir grains, green for home-made drinks and blue for industrial beverages.

To further explore the degree of diversity among the samples, an MDS/PCoA plot and a hierarchical clustering were generated based on OTUs that were taxonomically assigned at the family level. As shown in Figure 3A, two major clusters were observed in MDS/PCoA plot based on bacterial microbiota; the first, included the kefir grains 1G, 7G, and 8G, and the home-made drink 1D, and the second one contained the industrial beverages 2, 4, and 5. The specific grouping pattern of samples was also evident by the hierarchical clustering (Figure 3C). On the other hand, MDS/PCoA and hierarchical clustering based on the yeast/fungal communities grouped together the majority of the samples, i.e., all kefir grains (samples 1G, 6G, 7G, and 8G), home-made drinks 1D, 7D, and 8D and industrial drink 5, indicating that the majority of the samples analyzed shared a yeast/fungal microbiota at the family level (Figures 3B,D).

#### Phylogenetic Composition of the Bacterial Microbiota

To further analyze the microbial community structure of the samples, bacterial, and yeast/fungal OTUs were used to calculate relative abundances of taxa. Although V1–V3 region of the 16S rRNA gene offers the discriminatory power for bacterial identification at the species level (Johnson et al., 2019), due to the high-level similarity between closely related taxa, both bacterial and yeast/fungal microbiota of the samples were evaluated up to the genus level for a more accurate identification.

**FIGURE 4:**
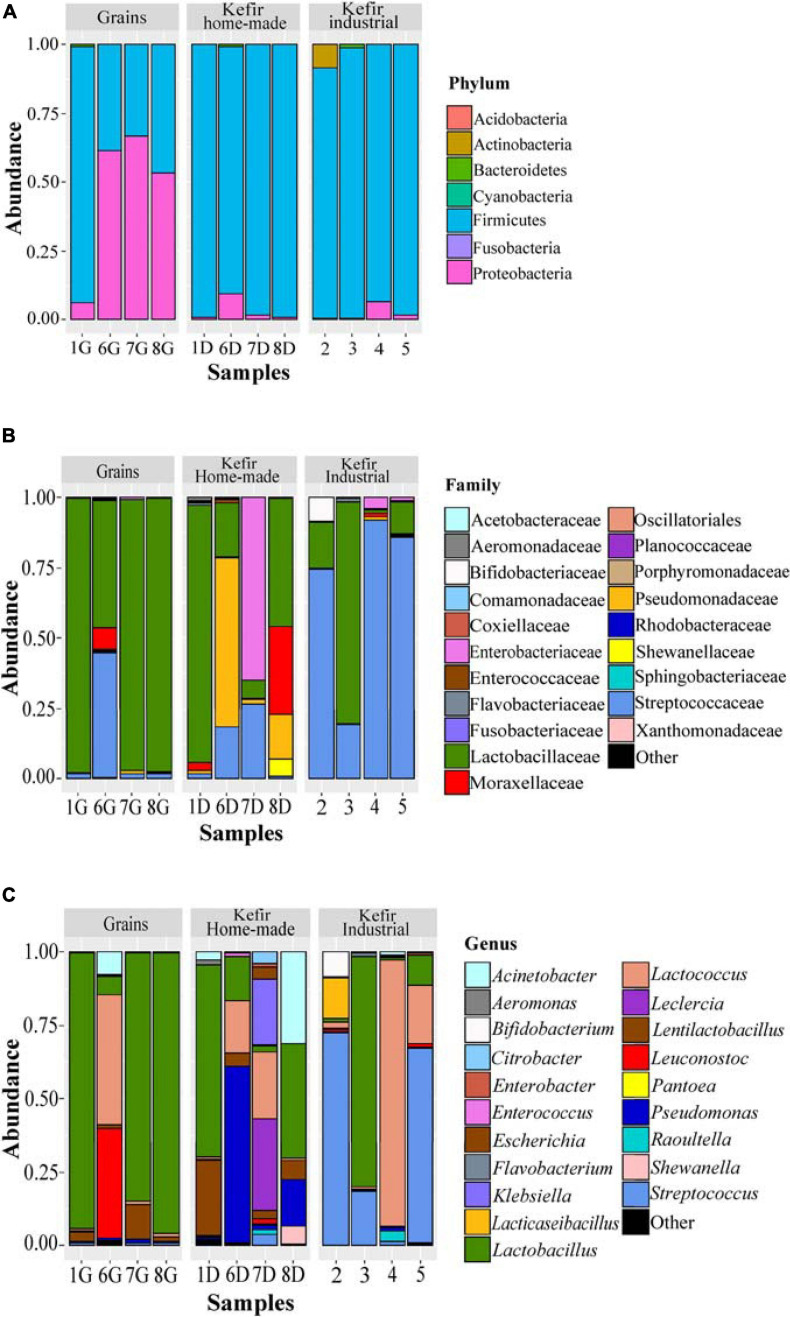
Composition plots of the relative abundances of the 20 most abundant bacterial OTUs taxonomically assigned at the phylum **(A)**, family **(B)**, and genus **(C)** level in kefir grains (samples 1G, 6G, 7G, and 8G), home-made (samples 1D, 6D, 7D, and 8D) and industrial (samples 2, 3, 4, and 5) drinks. Samples from each food group, i.e., kefir grains, home-made, and industrial drinks, are presented together in subpanels.

The bacterial microbiota of the 12 kefir grains and drinks analyzed, was covered by seven phyla, in which *Firmicutes* was the dominant with relative abundances ranging from 33.17 to 99.18%, followed by *Proteobacteria*, which was identified mainly in the home-made drinks 6D (61.35%), 7D (66.80%), and 8D (53.22%), and *Actinobacteria* found mostly in the industrial beverage 2 (8.57%) (Figure 4A and Supplementary Table S2A). Among *Firmicutes* and *Proteobacteria*, families *Lactobacillaceae and Streptococcaceae*, as well as *Pseudomonadaceae, Enterobacteriaceae*, and *Moraxellaceae*, respectively, were the most abundant (Figure 4B and Supplementary Table S2B). In details, the family *Lactobacillaceae* was the predominant in kefir grains 1G, 7G, and 8G, accounting for approximately 96% of the bacterial sequences. However, a more diverse microbiota was identified in grain sample 6G, which consisted of bacteria belonging to three main families, namely *Lactobacillaceae* (45.10%), *Streptococcaceae* (44.66%), and *Moraxellaceae* (7.87%). Although influenced by the microbial communities of kefir grains, the bacterial microbiota in respective home-made drinks was more diverse, apart from sample 1, in which the family *Lactobacillaceae* was predominant in both home-made kefir grains (1G: 97.30%) and drink (1D: 91.19%). Specifically, in the home-made drink 6D, bacterial family *Pseudomonadaceae* (60.31%) was found to be the most abundant, followed by *Lactobacillaceae* (19.20%) and *Streptococcaceae* (18.34%). In contrast, the family *Enterobacteriaceae* was the dominant (64.98%) in home-made drink 7D, followed by *Streptococcaceae* (26.57%) and *Lactobacillaceae* (6.60%). The dominance of *Enterobacteriaceae* family in sample 7D, together with either the absence or the relatively low abundances of this family in the other kefir grains and drink samples (< 4.05%), was in accordance with the results of the classical microbiological analysis, since *Enterobacteriaceae* counts were higher in sample 7D (6.5 log CFU mL^–1^) compared to the other samples (≤ 3.50 log CFU mL^–1^ or CFU g^–1^; Table 2). In addition, the microbial community structure of home-made drink 8D mainly consisted of bacteria belonging to the families *Lactobacillaceae* (45.68%), *Moraxellaceae* (31.20%), *Pseudomonadaceae* (15.62%), and *Shewanellaceae* (6.11%). As it was expected, bacterial microbiota of industrial beverages was less diverse compared to that of the home-made drinks, as also revealed by the alpha-diversity indices (Figure 2A). This is not surprising, as dairy industries use commercial starter cultures for a well-controlled fermentation that results in a standardized and safe final product. Bacterial communities identified in the industrial drinks (samples 2, 3, 4, and 5) belonged to three main families, namely *Streptococcaceae, Lactobacillaceae*, and *Bifidobacteriaceae*, with varying abundances among the samples. A closer look at the bacterial community structure classified at the genus level, revealed a similar distribution pattern to that observed at the family level, as one or two genera corresponded to all reads assigned to that family (Figure 4C and Supplementary Table S2C). Therefore, the genus *Lactobacillus* was the most abundant in kefir grains 1G (93.70%), 7G (84.48%), and 8G (95.51%), as well as in home-made drink 1D (65.18%). Furthermore, the genus *Lentilactobacillus* was also identified in relatively high abundances in samples 1G (3.37%), 7G (11.63%), 8G (1.51%), 1D (25.75%), 7D (2.65%), and 8D (6.51%). This was in consistent with the identification results obtained from the culture-dependent analysis, since *L. kefiri* was found to be the dominant species in these samples (Supplementary Table S1A). It is interesting though, that 7G was the only sample analyzed in which classical microbiological analysis identified two out of seven bacterial isolates as *S. warneri*, as mentioned above (Supplementary Table S1A). However, at the same time, none of the 16S sequences obtained from the metagenomics analysis for sample 7G was taxonomically assigned at the genus *Staphylococcus* (Supplementary Table S2C). Therefore, as *S. warneri* is a skin commensal of humans and animals (Kloos and Schleifer, 1975), the presence of the species in sample 7G could potentially be attributed to contamination during classical microbiological analysis. Furthermore, according to the results of the 16S metagenomics analysis, bacterial microbiota of kefir grains 6G was dominated by the genera *Lactococcus* (44.17%), *Leuconostoc* (37.15%), *Acinetobacter* (7.86%), and *Lactobacillus* (6.24%), while the respective home-made drink (sample 6D) by the *Pseudomonas* (60.31%), *Lactobacillus* (14.73%), and *Lactococcus* (17.96%) genera. On the other hand, classical microbiological analysis identified *L. mesenteroides* and *L. lactis* as the dominant species in samples 6G and 6D, respectively (Supplementary Table S1A). Moreover, *Lactobacillus* (39.02%), *Acinetobacter* (31.20%), *Pseudomonas* (15.62%), *Lentilactobacillus* (6.51%), and *Shewanella* (6.11%) were the most abundant genera found in home-made sample 8D. On the other hand, though, *Leclercia* (31.46%), *Klebsiella* (22.74%), *Lactococcus* (22.73%), *Lentilactobacillus* (2.65%), and *Lactobacillus* (2.22%) were the main genera of the home-made sample 7D bacterial microbiota, whereas *Enterobacter* species were mostly identified during microbiological analysis (∼56% of the bacterial isolates). However, the abundance of the genus *Enterobacter* was relatively low, i.e., 1.34% (Supplementary Table S2C). It should be noted, that the relatively high abundances of genera, such as *Pseudomonas, Leclercia, Klebsiella, Acinetobacter*, and *Shewanella*, in most of the home-made drinks analyzed, probably reflect the home-made production of these drinks.

In contrast, the bacterial communities of industrial beverages, namely 2, 3, 4, and 5, was dominated by common genera used as starter or adjunct cultures in dairy industry. More specifically, the genera *Streptococcus* (1.25–72.58%), *Lactococcus* (0.68–90.50%), *Lactobacillus* (1.06–78.52%), *Lacticaseibacillus* (0.03–14.06%), and *Bifidobacterium* (0.02–8.57%), were found to be the dominant ones with varying abundances among samples (Figure 4C and Supplementary Table S2C). As it was expected, the results of the classical microbiological analysis were consistent with those of the 16S metagenomics analysis. All the bacterial isolates from the industrial samples were identified as *S. thermophilus, L. delbrueckii* subsp. *bulgaricus, L. lactis, L. mesenteroides*, and *L. rhamnosus* (Supplementary Table S1A). Furthermore, as also mentioned above, even though high microbial counts were observed in GYP medium for all samples analyzed (6.11–8.67 log CFU mL^–1^ or CFU g^–1^; Table 2), Gram staining revealed that the majority of the isolates corresponded to either Gram-positive bacteria or yeasts. The almost absence of AAB using classical microbiological analysis was consistent with the results of the metagenomics analysis, since the family *Acetobacteraceae* was found only in two samples, namely 6G and 6D in trace levels, i.e., 0.09 and 0.25%, respectively (Supplementary Table S2B). However, several HTS studies have demonstrated that the genus *Acetobacter* is commonly found in both kefir grains and beverages as part of the subdominant bacterial microbiota (Leite et al., 2012; Gao et al., 2013; Marsh et al., 2013; Garofalo et al., 2015; Korsak et al., 2015; Walsh et al., 2016). Moreover, relatively high counts were also observed in KAA medium for samples 6G, 6D, 7D, 4, and 5 (Table 2). However, the genus *Enterococcus* was found in trace amounts (< 0.32%) in all samples except of sample 5 (1.07%; Supplementary Table S2C). Although not frequently, the genus *Enterococcus* has been identified in kefir grains and beverages as part of the subdominant microbiota (Dobson et al., 2011; Marsh et al., 2013; Nalbantoglu et al., 2014; Dertli and Con, 2017). In addition, the absence of *Listeria monocytogenes* and *Salmonella* spp. in all samples analyzed was confirmed by both classical microbiological and amplicon based-metagenomics analyses (Table 2 and Supplementary Tables S1A, S2C).

The identification of *Lactobacillaceae*/*Lactobacillus* as the dominant bacterial taxa in kefir grains and beverages has already been established by previous HTS studies. However, due to the new taxonomy of LAB, which was recently proposed by Zheng et al. (2020), the genus *Lentilactobacillus* should also be considered as part of the dominant bacteria microbiota of kefir grains and beverages since it includes, among others, the species *L. kefiri*. Apart from *Lactobacillus*, 16S rRNA metagenomics analysis of six Italian kefir grains, i.e., four home-made, one from the University of Perugia and one from a biotechnology company, revealed that although of different origins, the subdominant microbiota of all samples was in high concordance and consisted mainly of the genera *Acetobacter, Streptococcus*, and *Lactococcus* (Garofalo et al., 2015). Furthermore, HTS analysis of one kefir grain from the Ege University in Turkey and five home-made samples from different regions of the country revealed also highly similar communities dominated by *Lactobacillus* and several genera as part of the rest microbiota, such as *Pediococcus, Leuconostoc, Enterobacter*, and *Acinetobacter* (Nalbantoglu et al., 2014; Dertli and Con, 2017). However, there are also HTS studies that reported the identification of almost only *Lactobacillus* in kefir grains and beverages (Dobson et al., 2011; Leite et al., 2012; Zamberi et al., 2016). In addition, *Lactobacillus* and *Acetobacter* were found to be the most abundant genera identified in 28 kefir grains, either home-made or commercial, from eight distinct regions (Ireland, United Kingdom, United States, Spain, France, Italy, Canada, and Germany), whereas *Lactococcus*, *Acetobacter*, *Lactobacillus*, and *Leuconostoc* dominated the bacterial microbiota of the associated beverages (Marsh et al., 2013; Walsh et al., 2016). It should be noted though, that in a few studies, the genus *Lactobacillus* was not the predominant in kefir grains. According to Gao et al. (2013), HTS analysis of four Tibetan grains from different areas in China revealed that the genus *Lactococcus* was the most abundant (40.93–72.02%), while *Lactobacillus* was found to be among the subdominant microbiota along with *Acetobacter* and *Shewanella*. This was also the case for one commercial kefir grain sample from Belgium, in which the genus *Lactococcus* was the most abundant (93.7%), followed by *Leuconostoc* and *Lactobacillus* (Korsak et al., 2015). In the same study, four additional Belgian kefir grain samples were analyzed, i.e., two home-made and two obtained from the Ministry of Agriculture, and according to the 16S metagenomics analysis all samples were dominated by the genus *Lactobacillus* (> 85%), followed by *Gluconobacter*, *Lactococcus*, *Enterobacter*, and *Acetobacter.* Interestingly, although the respective kefir drinks comprised the same microbiota members, the relative abundances of the subdominant genera significantly increased in drinks compared to grains (Korsak et al., 2015). A similar trend was also observed in our samples, namely 7 and 8, since the bacterial microbiota of both 7G and 8G was dominated by the genus *Lactobacillus* (> 84%), while that of 7D and 8D was highly diverse including several genera, such as *Lactococcus*, *Klebsiella*, *Leclercia*, and *Acinetobacter* (Figure 4C and Supplementary Table S2C). To the best of our knowledge, this is the first study reporting the presence of *Acinetobacter* in kefir beverages. Although the genus is commonly present in soil and water (Baumann, 1968), *Acinetobacter* species have been also found in raw milk and cheeses, and contribute to the organoleptic characteristics of the final product due to their lipolytic and proteolytic activities (Fuka et al., 2010; Gurung et al., 2013; Pangallo et al., 2014). It should be noted however, that some *Acinetobacter* species, such as *Acinetobacter baumannii*, are considered as opportunistic pathogens mainly associated with hospital-acquired infections (de Amorim and Nascimento, 2017).

#### Phylogenetic Composition of the Yeasts/Fungal Microbiota

**FIGURE 5:**
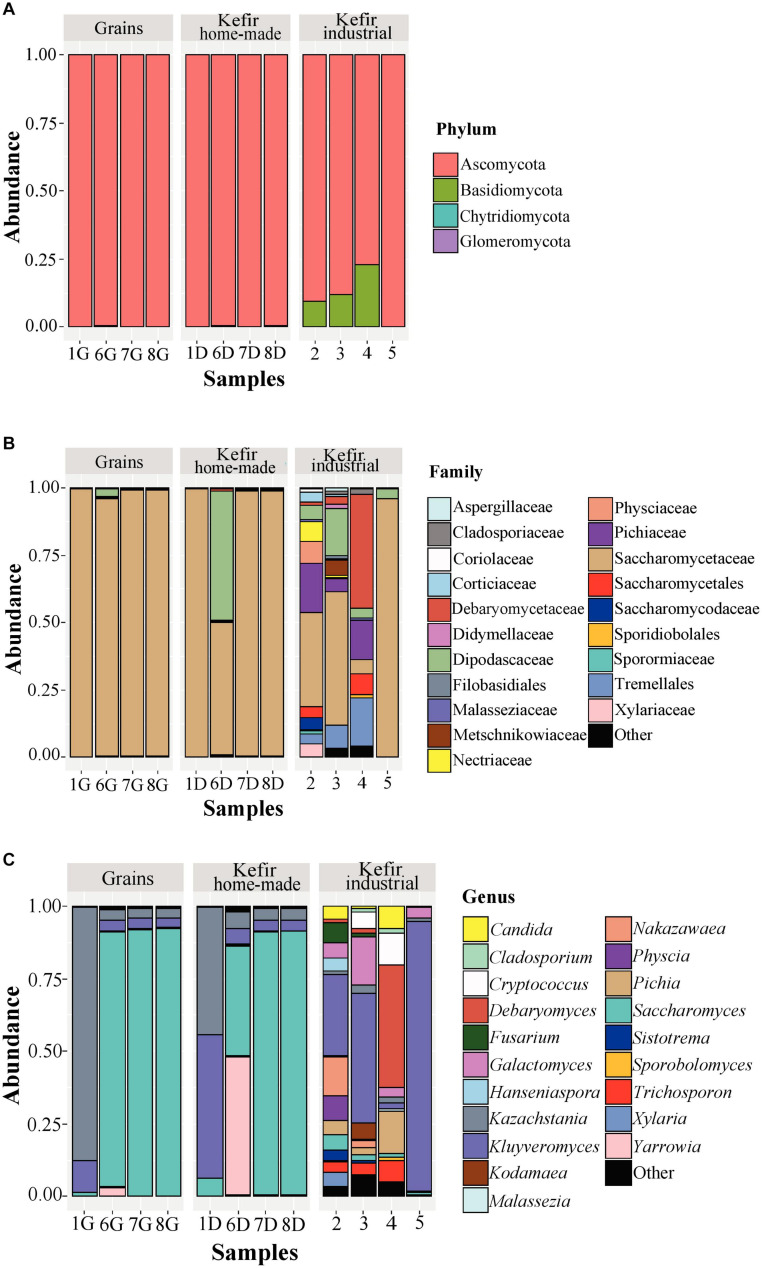
Composition plots of the relative abundances of the 20 most abundant yeast/fungal OTUs taxonomically assigned at the phylum **(A)**, family **(B)**, and genus **(C)** level in kefir grains (samples 1G, 6G, 7G, and 8G), home-made (samples 1D, 6D, 7D, and 8D) and industrial (samples 2, 3, 4, and 5) drinks. Samples from each food group, i.e., kefir grains, home-made, and industrial drinks, are presented together in subpanels.

The yeast/fungal microbiota of kefir grains and beverages was characterized by a high level of *Ascomycota* phylum with relative abundances ranging from 79.18 to 99.93%. It should be noted however, that yeasts/fungi belonging to the phylum *Basidiomycota* were found to be present in relatively high abundances in industrial beverages 2 (9.66%), 3 (11.94%), and 4 (12.82%) (Figure 5A and Supplementary Table S3A). Compared to bacteria, yeasts/fungal microbiota classified at the family level was less diverse in kefir grains and home-made drinks, with *Saccharomycetaceae* being the dominant family (> 95.00%) in all samples but 6D, in which *Saccharomycetaceae* and *Dipodascaceae* were identified in similar abundances, i.e., 49.12 and 48.17%, respectively (Figure 5B and Supplementary Table S3B). The identification of *Dipodascaceae* family in drink sample 6D, could be explained by the proportion of the same family in kefir grain 6G (2.81%). However, based on the two proportions, i.e., 48.17 and 2.81% for samples 6D and 6G, respectively, we can assume that the milk environment favored the abundance of yeasts/fungi belonging to the family *Dipodascaceae*. Moreover, as also revealed by the alpha-diversity indices (Figure 2B), the diversity of industrial beverages was significantly higher than that of home-made ones (Figure 5B). It was interesting though, that this was not the case for the industrial drink sample 5, in which *Saccharomycetaceae* was the predominant family at 95.76% abundance. In the industrial drink sample 2, *Saccharomycetaceae* (34.78%), *Pichiaceae* (18.16%), *Physciaceae* (8.48%), and *Nectriaceae* (6.95%) were the main families identified, while yeast/fungal microbiota of the industrial sample 3 was dominated by the families *Saccharomycetaceae* (49.57%), *Dipodascaceae* (17.44%), and *Tremellales* (8.64%). The only sample that the family *Saccharomycetaceae* was not the dominant, was the industrial drink 4. In this sample, *Debaryomycetaceae* was the most abundant (42.47%), followed by *Tremellales* (18.11%), *Pichiaceae* (14.80%), *Saccharomycetales* (7.53%), and *Saccharomycetaceae* (5.34%). The dominance of the family *Debaryomycetaceae* in sample 4 could be explained by the use of eXact KEFIR1 culture for milk fermentation, which contains five bacterial species along with the yeast species *D. hansenii*. At the genus level, yeast/fungal communities of kefir grains 7G and 8G were almost identical to those of the respective home-made drinks, namely 7D and 8D, and dominated by the genus *Saccharomyces* (> 90%), followed by *Kazachstania* and *Kluyveromyces* with abundances for both genera below 4% (Figure 5C and Supplementary Table S3C). Similar were also the yeast/fungal microbiota profiles between samples 1G and 1D, although with different abundances. In detail, *Kazachstania* (87.40%) and *Kluyveromyces* (11.12%) were found to be the most abundant genera in grain sample 1G, while in home-made drink 1D, *Kluyveromyces* (49.58%) was the dominant genus, followed by *Kazachstania* (43.74%) and *Saccharomyces* (6.08%). The yeast/fungal microbiota of kefir grain 6G and home-made drink 6D had an analogous to the bacterial microbiota trend. Although the genus *Yarrowia* was found in trace levels in sample 6G, the abundance of this genus in sample 6D was significantly higher, i.e., 47.67%, and along with *Saccharomyces* (38.03%), were the most abundant genera sample 6D. In contrast, the yeast/fungal microbiota of sample 6G was dominated by the genus *Saccharomyces* (87.99%). The trend obtained by the ITS metagenomics analysis, i.e., similar yeast/fungal microbiota profiles between each grain-drink pair was also revealed by the classical microbiological analysis. *K. marxianus* and *K. turicensis* were the only species identified in both samples 1G and 1D, *S. cerevisiae* and *Y. lipolytica* in samples 6G and 6D and *S. cerevisiae* in samples 7G and 7D as well as 8G and 8D (Supplementary Table S1B). Concerning the industrial beverages, sample 5 was the least diverse, as also observed by the classification at the family level, and was dominated by the genus *Kluyveromyces* (93.57%). Although the industrial samples 2 and 3 were more diverse than sample 5, the genus *Kluyveromyces* was also found to be the most abundant, followed by several other genera, such as *Nakazawaea, Physcia, Galactomyces, Fusarium*, and *Saccharomyces*, with abundances varying between the two samples (Supplementary Table S3C). Similarly to the identification results at the family level, yeast/fungal community structure in industrial beverage 4 was dominated by the genus *Debaryomyces* (42,47%), followed by *Pichia* (14,68%) and *Cryptococcus* (10,89%). The results from the ITS metagenomics analysis were in accordance with those obtained by the classical microbiological analysis concerning samples 4 and 5. *P. kudriavzevii, D. hansenii*, and *Geotrichum candidum/Galactomyces candidum* as well as *K. marxianus* and *Geotrichum candidum/Galalactomyces candidum* were the yeast species identified in samples 4 and 5, respectively (Supplementary Table S1B). It is interesting however, that although high abundances of yeast/fungal genera revealed by the ITS metagenomics analysis for samples 2 and 3, yeasts were not detected during the microbiological analysis. The absence of yeast counts in samples 2 and 3 may imply that their abundances derived from the amplification of dead or compromised cells, which is a well-known disadvantage of DNA-based metagenomics techniques (De Filippis et al., 2017).

Our study is among the few elucidating the yeast/fungal microbiota of kefir grains and beverages using metagenomics analysis. Although yeasts/fungi also contribute to the organoleptic characteristics of several dairy products, including kefir, the number of HTS studies describing the bacterial communities is significantly higher than that of yeasts/fungi (De Filippis et al., 2017). The dominance of *Saccharomyces, Kazachstania*, and *Kluyveromyces* in our kefir grains (Figure 5C) was in accordance with the results of the other HTS studies. Specifically, the yeast/fungal microbiota of five kefir grains of different origins revealed a similar profile among the samples, with *Kazachstania* and *Kluyveromyces* being the most abundant genera identified (Korsak et al., 2015). In contrast, a different yeast/fungal microbial profile was found between home-made and academic/commercial samples. The latter were dominated almost exclusively by the genera *Kazachstania* and *Dekkera*, while the community structure of home-made samples was more diverse containing also the genera *Saccharomyces* and *Hanseniaspora* with varying abundances depending on the sample analyzed (Garofalo et al., 2015). An even more diverse microbiota was found in four home-made samples from different regions of Turkey, composed by members of the family *Dipodascaceae* and the genera *Saccharomyces, Kazachstania, Candida, Issatchenkia*, and *Rhodotorula* in lower abundances (Dertli and Con, 2017). The yeast/fungal communities of our home-made beverages analyzed were also dominated by the genera *Saccharomyces*, *Kazachstania*, and *Kluyveromyces*, as in the case of kefir grains. However, in sample 6D, *Yarrowia* was also identified (Figure 5C). On the other hand, the yeast/fungal microbiota of industrial samples was significantly more diverse than that of the home-made ones, as also revealed by the alpha-diversity indices (Figure 2B), and mainly composed by the genera *Kluyveromyces, Debaryomyces, Galactomyces, Cryptococcus, Pichia*, and *Nakazawaea* (Figure 5C). Interestingly, according to the study of Marsh et al. (2013), the identified yeast/fungal communities of 25 kefir grains (home-made and industrial) obtained from eight geographically distinct regions and the respective drinks were, in high concordance, dominated by the genera *Kazachstania*, *Naumovozyma*, and *Kluyveromyces*. This was also the case in the study of Walsh et al. (2016), in which *Saccharomyces* and *Kazachstania* were the most abundant genera found in three kefir grains from France, Ireland, and the United Kingdom and their drinks accounted for > 99% of the ITS sequences. Eventually, the relatively few reports regarding the yeast/fungal microbiota of both kefir grains and beverages, highlight the need of more HTS studies to unravel the microbial composition of these ecosystems.

## Conclusion

The concurrent employment of a culture-dependent approach and amplicon-based metagenomics analysis unraveled, in a consistent way, the rich diversity of the microbiota of home-made and industrial kefir samples produced in Greece. Culturing methods enabled not only the estimation of the viable counts within various microbial groups but also the isolation of microorganisms for potential future applications in kefir production. Identification of isolated strains revealed that certain bacterial and yeast/fungal genera were mainly associated with either the home-made or the industrial samples. The presence of *Enterobacteriaceae* in both home-made and industrial samples along with the identification of (opportunistic) pathogens in one home-made sample, underlines the necessity of employing good hygiene practices in kefir production both at home and industrial level.

Interestingly, in the home-made samples the dominant genera of grains were different from those of the respective drinks. Overall, the in-depth study of kefir microbiota can help us recognize and possibly tune microbial activities to improve sensory characteristics and product quality and safety.

## Data Availability Statement

The datasets presented in this study can be found in online repositories. The names of the repository/repositories and accession number(s) can be found below: https://www.ebi.ac.uk/ena, PRJEB37688.

## Author Contributions

MK supervised the total DNA extraction for metagenomics analysis, performed the bioinformatics analysis of the amplicon-based metagenomics data and participated in the writing and review of the manuscript. AG performed the classical microbiological analysis and the total DNA extraction for the metagenomics analysis and participated in the writing of the manuscript. ET supervised the classical microbiological and metagenomics analyses and participated in the writing and review of the manuscript. MG conceived the project, supervised the classical microbiological analysis, and participated in the writing of the manuscript. All authors read and approved the final manuscript.

## Conflict of Interest

The authors declare that the research was conducted in the absence of any commercial or financial relationships that could be construed as a potential conflict of interest.

## Supplementary Material

Supplementary Figure 1 Rep-PCR fingerprinting of bacterial isolates using the BOXAIR primer. Image analysis was performed using BioNumerics v. 6.0. Arrows denote isolates, which were selected for 16S rRNA gene sequencing.

Supplementary Figure 2 Rep-PCR fingerprinting of yeast isolates using the (GTG)_5_ primer. Image analysis was performed using BioNumerics v. 6.0. Arrows denote isolates, which were selected for ITS DNA region sequencing.

Supplementary Table 1 Identification of bacterial isolates using classical microbiological analysis **(A)**. Identification of yeasts isolates using classical microbiological analysis **(B)**.

Supplementary Table 2 Relative abundance of bacterial phyla identified in kefir grains, homemade, and industrial drinks **(A)**. Relative abundance of bacterial families identified in kefir grains, homemade, and industrial drinks **(B)**. Relative abundance of bacterial genera identified in kefir grains, homemade and industrial drinks **(C)**.

Supplementary Table 3 Relative abundance of yeast/fungal phyla identified in kefir grains, homemade, and industrial drinks **(A)**. Relative abundance of yeast/fungal families identified in kefir grains, homemade, and industrial drinks **(B)**. Relative abundance of yeasts/fungal genera identified in kefir grains, homemade, and industrial drinks **(C)**.
