# Supplementary material for: Zooming Into the Microbiota of Home-Made and Industrial Kefir Produced in Greece Using Classical Microbiological and Amplicon-Based Metagenomics Analyses
Source: Front Microbiol. 2021 Jan 28;12:621069. doi: 10.3389/fmicb.2021.621069 (PMC7876260; doi:10.3389/fmicb.2021.621069)
Supplement: Supplementary Figure 1 — Rep-PCR fingerprinting of bacterial isolates using the BOXAIR primer. Image analysis was performed using BioNumerics v. 6.0. Arrows denote isolates, which were selected for 16S rRNA gene sequencing. [file Presentation_1.PPT]

## Slide 1
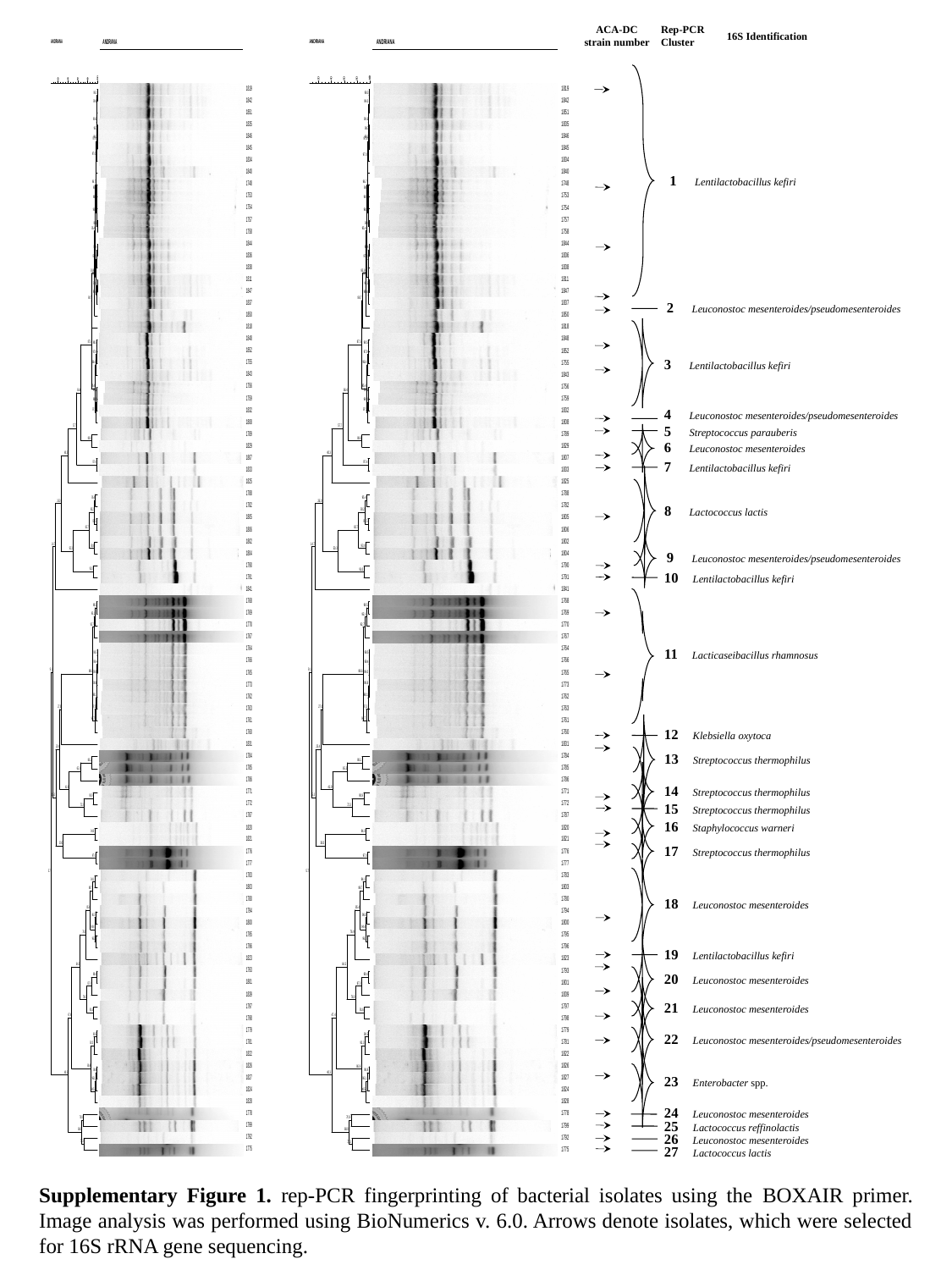

ACA-DC strain number
Rep-PCR Cluster
16S Identification
1 Lentilactobacillus kefiri
2 Leuconostoc mesenteroides/pseudomesenteroides
3 Lentilactobacillus kefiri
4 Leuconostoc mesenteroides/pseudomesenteroides
5 Streptococcus parauberis
6 Leuconostoc mesenteroides
7 Lentilactobacillus kefiri
8 Lactococcus lactis
9 Leuconostoc mesenteroides/pseudomesenteroides
10 Lentilactobacillus kefiri
11 Lacticaseibacillus rhamnosus
12 Klebsiella oxytoca
13 Streptococcus thermophilus
14 Streptococcus thermophilus
15 Streptococcus thermophilus
16 Staphylococcus warneri
17 Streptococcus thermophilus
18 Leuconostoc mesenteroides
19 Lentilactobacillus kefiri
20 Leuconostoc mesenteroides
21 Leuconostoc mesenteroides
22 Leuconostoc mesenteroides/pseudomesenteroides
23 Enterobacter spp.
24 Leuconostoc mesenteroides
25 Lactococcus reffinolactis
26 Leuconostoc mesenteroides
27 Lactococcus lactis
Supplementary Figure 1. rep-PCR fingerprinting of bacterial isolates using the BOXAIR primer. Image analysis was performed using BioNumerics v. 6.0. Arrows denote isolates, which were selected for 16S rRNA gene sequencing.

## Slide 2
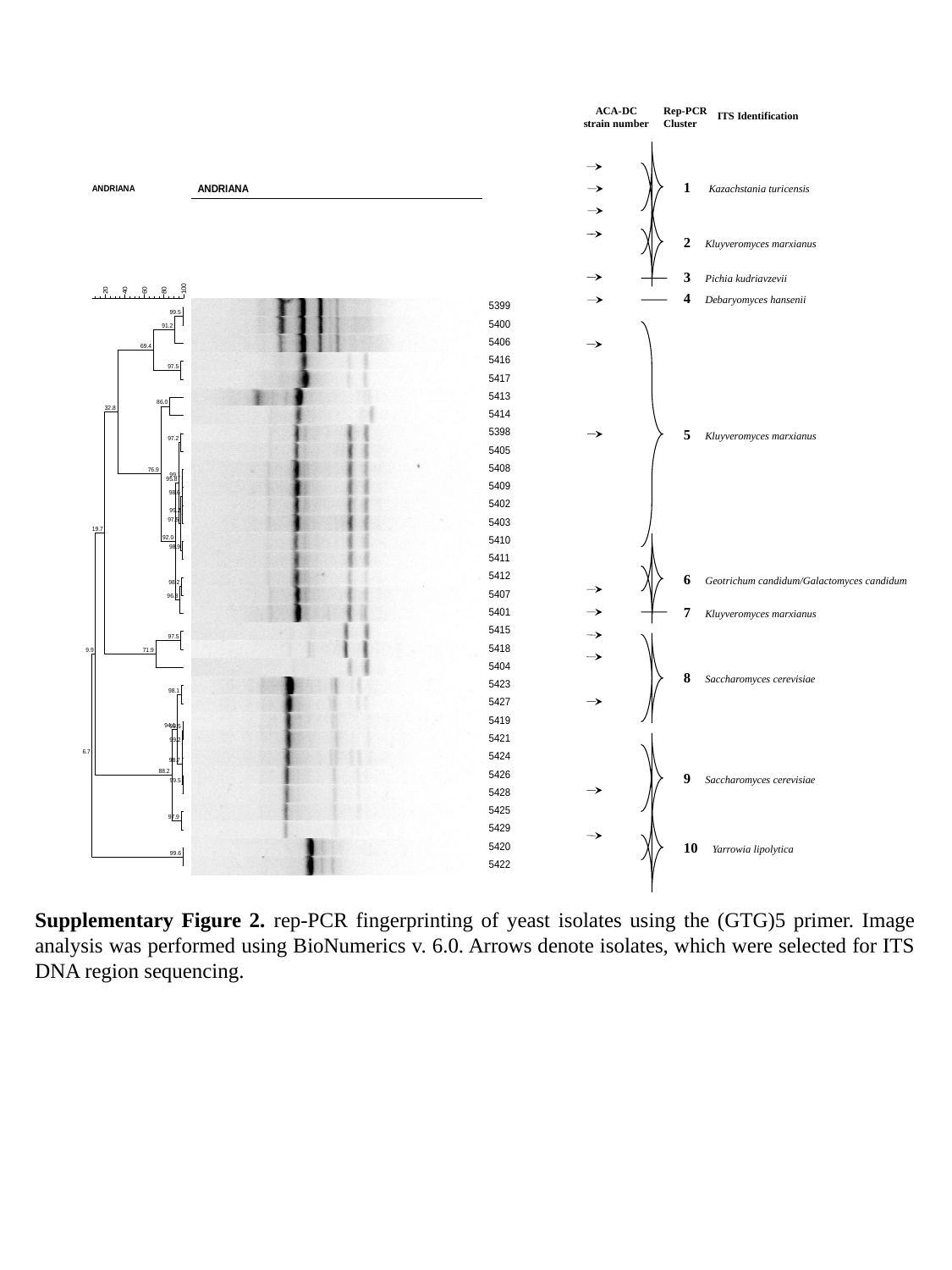

ACA-DC strain number
Rep-PCR Cluster
ITS Identification
1 Kazachstania turicensis
2 Kluyveromyces marxianus
3 Pichia kudriavzevii
4 Debaryomyces hansenii
5 Kluyveromyces marxianus
6 Geotrichum candidum/Galactomyces candidum
7 Kluyveromyces marxianus
8 Saccharomyces cerevisiae
9 Saccharomyces cerevisiae
10 Yarrowia lipolytica
Supplementary Figure 2. rep-PCR fingerprinting of yeast isolates using the (GTG)5 primer. Image analysis was performed using BioNumerics v. 6.0. Arrows denote isolates, which were selected for ITS DNA region sequencing.
